# A Patient With Charcot-Marie-Tooth Disease Type 4C (CMT4C) Presenting With Muscle Fasciculations and Motor Neuropathy

**DOI:** 10.7759/cureus.57550

**Published:** 2024-04-03

**Authors:** Leema Reddy Peddareddygari, Raji P Grewal

**Affiliations:** 1 Research and Development, Dynamic Biologics Inc., Monmouth Junction, USA; 2 Neurosciences, Capital Health Institute for Neurosciences, Bordentown, USA

**Keywords:** motor neuropathy, fasciculations, rare variants, sh3tc2 gene, charcot-marie-tooth disease type 4c

## Abstract

We report an unusual patient who, at age 47 years, had presented with complaints of muscle fasciculations. After neurological examination and electromyogram testing, he was diagnosed with motor neuropathy. Over the next 10 years, in addition to fasciculations, he developed numbness in his feet without any other symptoms. His current neurological examination at age 57 years was normal, except for mildly decreased light touch in the anterior portion of both feet. The nerve conduction studies performed repeatedly showed sensorimotor polyneuropathy with demyelination features. Blood tests, including anti-ganglioside antibodies, were normal. Genetic testing revealed two rare variants in *trans* in the *SH3 domain and tetratricopeptide repeats 2*gene, c.3413 G>A p.(S1138N) and c.3269 C>G p.(A1090G). Protein modeling suggests that these are disease-producing mutations and likely the cause of the neuropathy of our patient. Our study expands the clinical and genetic spectrum of patients with Charcot-Marie-Tooth disease type 4C.

## Introduction

Charcot-Marie-Tooth (CMT) disease refers to a group of hereditary motor sensory neuropathies. In the past, CMT was classified by mode of transmission and whether the neuropathy was axonal or demyelinating. More recently, the classification is gene-based and mutations have been described in more than 80 different genes [1].

CMT can follow an autosomal dominant, recessive, or sex-linked pattern of inheritance. CMT disease type 4C (CMT4C) typically follows an autosomal recessive (AR) pattern of inheritance. It is caused by homozygous or compound heterozygous mutation in the *SH3 domain and tetratricopeptide repeats 2 (SH3TC2) *gene.

In investigations of patients with CMT, CMT4C is not common, with a frequency of 1.5-4%, which is partially a reflection of the population studied [2,3]. However, it is one of the more common causes of AR-CMT, with a frequency of up to 20% of AR demyelinating forms of CMT [1].

A broad range of clinical presentations have been reported, and, in some families, haploinsufficiency has been associated with mild clinical phenotypes [4-6].

In a recent publication specifically describing this form of CMT, the most common presenting symptoms and signs include walking difficulties, scoliosis, and foot deformities [7]. We report an unusual patient with CMT4C who initially presented with muscle fasciculations and motor neuropathy.

## Case presentation

 A 57-year-old male patient was referred for a second opinion for the diagnosis of motor neuropathy. Neurological history reveals that, 10 years earlier, at age 47 years, he had noted fasciculations in the muscles of his calves and feet. At that time, he had no sensory complaints in his feet. The patient had a paternal aunt who was diagnosed with amyotrophic lateral sclerosis (ALS), and he was concerned about having the same diagnosis. Consequently, he obtained an opinion from a neurologist with expertise in motor neuron disease at a university center. On examination at age 47 years, he was noted to have normal power bulk and tone in all limbs. His sensory examination was normal. His reflexes were +2 testing the brachioradialis, biceps, triceps, patellae, and ankles with flexor plantar reflexes. Further testing was done at his initial presentation, including an electromyogram (EMG), which demonstrated markedly reduced response amplitudes in the tibial nerves with normal conduction velocities and prolonged F-wave latencies (Table 1).

**Table 1:** Findings from the initial nerve conduction study of the patient. L - Left, R - Right, NR - No response, AE - Above elbow, BE - Below elbow, P - Stimulation at popliteal fossa

Nerve	Distal latencies (ms)	Response amplitude (mV)	Conduction velocity (m/s)	F-wave latency (ms)
Motor				
L. Median	4.2	6.9	50	32
		7.1		
L. Ulnar	3.0	9.1	57	32
		7.7 (BE)	59	
		6.8 (AE)		
R. Peroneal	4.4	4.4	40	NR
		3.3		
L. Peroneal	6.4	4.2	40	60.1
		4.2		
R. Tibial	4.8	1.2	40	69.9
		1.2		
L. Tibial	4.8	1.3	40	72.1
		1.1 (P)		
Sensory	Distance (cm)			
R. Sural	14	10	40	
L. Sural	14	10	40	
L. Median	13	19	57	
L. Ulnar	11	16	50	
L. Radial	10	23	50	
R. Peroneal	14	10	45	
L. Peroneal	14	10	40	

The parameters in both peroneal nerves were normal, except that no F-wave latency was obtained on the right, whereas it was normal on the left. All motor parameters, including F-wave latencies, were normal in the median and ulnar nerves. Sensory nerve action potentials were normal in the median, ulnar, radial, sural and superficial peroneal nerves. No other denervation potentials were observed on needle EMG. No abnormalities were detected in sampling other muscles of the legs or his arms.

The results of routine blood work, including a complete blood count (CBC) and differential, comprehensive metabolic panel, serum protein electrophoresis and immunofixation, and B12 levels, were normal. In addition, testing antibodies against anti-myelin-associated glycoprotein (MAG), ganglioside (Gm1) antibodies (IgG and IgM), and other gangliosides were negative. The following genes established to cause familial ALS and those that were commercially available at that time were tested and did not reveal any pathogenic mutations: *superoxide dismutase type 1, TAR DNA-binding protein, angiogenin, FIG4 phosphoinositide 5-phosphatase, *and *fused in sarcoma (FUS) RNA-binding protein*. A magnetic resonance imaging of his lumbosacral spine disclosed no significant abnormalities that could account for his symptoms. The patient was reassured that he did not have ALS and was diagnosed with a distal motor neuropathy with no evidence of a diffuse neurogenic process.

Over the next 10 years, the patient continued to experience fasciculations in the muscles of his feet and lower legs but was able to exercise vigorously, with no complaints of weakness or fatigue. Although the exact onset is not clear to the patient, at least five years later, he developed numbness in the anterior portion of both feet, which slowly progressed. This was not associated with back pain, numbness, or tingling elsewhere and with no other neurological symptoms. Neurological re-examination revealed a normal mental status, cranial nerve examination (cranial nerves 2-12), and tests of cerebellar function. Power testing revealed Grade 5/5 (medical research council scale) testing both proximal and distal musculature including foot dorsiflexion, inversion, and eversion. He could stand and walk on his heels and toes. Sensory testing revealed a mild decrease in light touch in the anterior portion of his feet with preservation of proprioception and vibratory sense. Reflex testing were +2 at the biceps, triceps, brachioradialis, and patella and 1+ at the ankles with flexor plantar responses. His gait and station were normal, and his Romberg test was normal. He could perform a tandem walk without difficulty and had no foot deformity or scoliosis.

An EMG was repeated and showed that sensory responses in the sural and superficial peroneal nerves were not obtained (Table 2).

**Table 2:** Findings from the repeat nerve conduction study of the patient. L - Left, R - Right, NR - No response, F - Stimulation at the fibular head

Nerve	Distal latencies (ms)	Response amplitude (mV)	Conduction velocity (m/s)	F-wave latency (ms)
Motor				
R. Peroneal	9.3	0.9	33	NR
		0.6 (F)		
L. Peroneal	13.3	0.3	33	NR
		0.3 (F)		
R. Tibial	NR	NR		NR
L. Tibial	NR	NR		NR
Sensory				
R. Sural	NR			
L. Sural	NR			
R. Peroneal	NR			
L. Peroneal	NR			

In addition, with maximal stimulation, recording the abductor hallucis muscle, no evoked responses were obtained in either tibial nerve. There were reduced response amplitudes in both peroneal nerves with demyelinating features (prolonged distal latencies and slowing of the motor conduction velocities). Needle EMG showed fasciculation potentials with rare positive sharp waves in muscles sampled below the knees (medial gastrocnemius, tibialis anterior). Overall, the study is consistent with a demyelination length-dependent neuropathy affecting both motor and sensory fibers and with minimal secondary axonal features.

As noted, the family history was significant for a paternal aunt with ALS but no other neurological disorders. His parents and two children do not suffer from a neurological disorder.

The following investigations were normal or negative: CBC and differential, vitamin B1, B12, E, and B6, hemoglobin A1c, angiotensin-converting enzyme, serology testing (hepatitis C, human T-lymphotropic virus 1 and 2, human immunodeficiency virus, Lyme), creatine phosphokinase, aldolase, C-reactive protein, serum protein electrophoresis and immunofixation, intrinsic factor antibodies, antigliadin antibodies, transglutaminase antibodies, anti-GM1 autoantibodies, anti-MAG autoantibodies, and a two-hour glucose tolerance test.

Genetic analysis

Following Institutional Review Board protocols and procedures, whole exome sequencing was done commercially, with a panel testing more than 80 genes established to cause neuropathy. A pathogenic variant was detected in the *polynucleotide kinase-phosphatase *gene, rs199919568, a splice donor variant, c.1029+2T>C in intron 11 (NM_007254.2). Mutations in this gene can cause CMT type 2, but the mode of transmission is autosomal recessive, and a second mutation was not detected. Another variant was found in the *lamin* *A* gene, c.1658_1659delAA (Q553Rfs*79), and, although mutations in this gene can cause CMT type 2B1, this is an autosomal recessive condition, and a second mutation was not identified.

Two variants were detected in the *SH3TC2* gene, c.3413 G>A p.(S1138N) and c.3269 C>G p.(A1090G). To investigate whether these variants were in cis or trans, a daughter agreed to participate in this study, and the results indicate that she carries only the c.3269 C>G p.(A1090G) variant. These results confirm that the variants are in trans in the patient. The first variant, c.3413 G>A p.S1138N, exon 11, rs150805608, has a frequency of T=0.00007070 (20/282882) in gnomAD (https://gnomad.broadinstitute.org/variant/5-148388479-C-T?dataset=gnomad_r2_1) [8]. This is a missense mutation that causes an amino acid change from serine to asparagine. Protein modeling with structural interaction fingerprint (SIFT) [9], PolyPhen-2 [10], and Align-GVGD [11] suggests that this change is likely to be tolerated. However, analysis with the mutation taster tool [12] indicates that this variant can affect protein structure or function by splice site changes. The second variant identified in the *SH3TC2 *gene in our patient was c.3269 C>G p.A1090G, exon 10, rs374853461, and has a frequency of C=0.00004953 (14/282678) in genomAD (https://gnomad.broadinstitute.org/variant/5-148389891-G-C?dataset=gnomad_r2_1) [8]. In this variant, there is a change from alanine to glycine. Protein modeling with SIFT, PolyPhen-2, Align-GVGD, and mutation taster predicts this variant as disease producing by causing a deleterious effect on protein structure or/and function.

## Discussion

Genetic testing identified two variants in the *SH3TC2* gene in our patient, and both variants are very rare and have been reported in heterozygous state only. The S1138N variant has been observed in an individual with CMT disease, although a second pathogenic variant was not identified [3]. This variant is reported in the ClinVar database and is classified as a variant of unknown significance. It is located on exon 11 that has been observed to be a hotspot for mutations in CMT4C [1]. Although the A1090G variant has not been reported in the ClinVar database, it has been noted in the gnomAD database in the heterozygous state. It is highly likely that these two *SH3TC2* mutations, S1138N and A1090G, are the cause of the neuropathy in our patient confirming that he has CMT4C.

Peripheral neuropathy is a common disorder that has been reported in up to 8% of the general population over age 65 years [13]. When common causes such as diabetes mellitus are excluded, a significant proportion of patients are diagnosed as “idiopathic”. In a report of 284 patients who were referred to a neuromuscular center with the diagnosis of idiopathic neuropathy, even after extensive investigations, no diagnosis was achieved in 32.7% [14]. This was a retrospective analysis of patients evaluated from 2002 to 2012, in a period when commercial genetic testing was difficult to obtain. Interestingly, a genetic diagnosis was made in only one patient. In 2014, a study reported the results of 17,880 patients with neuropathy who were referred to a genetic testing company, and at this time, sequencing technology had improved and was much less expensive [15]. In this sample, genetic abnormalities were found in 3,312 patients, and of these, CMT4C was diagnosed in 0.8%. In a more recent large-scale study of patients suspected to have genetic neuropathy, 3.3% of 1,515 patients were identified with mutations in the SH3TC2 gene [16]. The increase in the identification of patients with CMT4C is, in part, due to the improvements in sequencing technology. Nevertheless, CMT4C remains an uncommon condition and an infrequent cause of CMT.

CMT4C has a variable clinical presentation from severe-to-mild phenotypes, with an age of onset ranging from one to 73 years of age. The adult-onset form usually presents with foot deformities and scoliosis [1]. Most patients with CMT4C exhibit the typical features of neuropathy including distal weakness and numbness associated with atrophy and loss of reflexes. However, our patient presented with muscle fasciculations and motor neuropathy at 47 years of age.

He initially sought neurological consultation for muscle fasciculations. Interestingly, although he had normal reflexes, normal sensation, and power in his legs, the initial EMG did show evidence of a distal neuropathy with exclusive involvement of the motor fibers of the tibial nerves and muscle fasciculations. In the initial EMG, the prolonged F-wave latencies are a demyelination feature and consistent with CMT4C although preferential motor involvement is unusual (Table 1). Muscle fasciculations are not an unusual feature of motor neuropathy [17]. It is of note that the repeat EMG done 10 years later showed an evolution of his neuropathy (Table 2). There was now further evidence of a more diffuse neuropathy with sensory and motor nerve fiber involvement with demyelinating and secondary axonal features.

## Conclusions

Overall, this patient’s phenotype is mild, and, even at age 57 years, he had minimal sensory loss in the feet without clinical motor deficits. Our study expands the clinical spectrum of CMT4C patients who can present with fasciculations and motor neuropathy. It also demonstrates the importance of follow-up and re-examination of patients with muscle fasciculations. Furthermore, the study expands the mutation spectrum that can cause CMT4C. A specific diagnosis is important as it facilitates genetic counseling and the participation of patients in future clinical trials for the treatment of this genetic neuropathy.
